# Kinetic studies on the removal of phenol by MBBR from saline wastewater

**DOI:** 10.1186/s40201-017-0284-0

**Published:** 2017-10-26

**Authors:** Mehdi Ahmadi, Neamat Jaafarzadeh, Zeinab Ghaed Rahmat, Ali Akbar Babaei, Nadali Alavi, Zeinab Baboli, Mehdi Vosoughi Niri

**Affiliations:** 1 Environmental Technologies Research Center, Ahvaz Jundishapur University of Medical Sciences, Ahvaz, Iran; 2 Department of Environmental Health Engineering, Ahvaz Jundishapur University of Medical Sciences, Ahvaz, Iran; 3 Department of Environmental Health Engineering, Behbahan Faculty of Medical Sciences, Behbahan, Iran; 4 Ahvaz Jundishapur University of Medical Sciences, Ahvaz, Iran; 5 Environmental and Occupational Hazards Control Research Center, Shahid Beheshti University of Medical Sciences, Tehran, Iran; 6 Department of Environmental Health Engineering, School of Public Health, Shahid Beheshti University of Medical Sciences, Tehran, Iran; 7 Department of Environmental Health Engineering, Faculty of Health, Ardabil University of Medical Sciences, Ardabil, Iran

**Keywords:** Saline wastewater, Phenol, MBBR, Kinetic model, Biological process

## Abstract

**Background:**

Phenols are chemical compounds which are included in the high priority of pollutants by environmental protection agency (USEPA). The presence of high concentrations of phenols in wastewaters like oil refineries, petrochemical plants, olive oil, pesticide production and oil field operations contain high soluble solids (TDS) and in an olive oil plant, wastewater is acidic, high salty and phenol concentrations are in the range of 0.1- 1%.

**Methods:**

Kinetic parameters were calculated according to Monod, Modified Stover- Kincannon, Hamoda and Haldane models. The influence of different initial phenol concentrations on the biodegradation rate was performed. The concentrations of phenol varied from 0 to 500 mg*/*l.

**Results:**

The value of K_i_ in saline phenolic wastewater in attached growth systems was higher than suspended growth systems that represented a higher phenol inhibition in suspended growth systems. It was obvious that the best model fitting the obtained data are Hamoda model and the Modified Stover-Kincannon model, having highest *R* ^*2*^ values of 0.991 and 1, respectively. The value of K_i_ in saline phenolic wastewater in attached growth system was higher than suspended growth systems which represented a higher phenol inhibition in suspended growth systems.

**Conclusions:**

Hamoda model and the Modified Stover-Kincannon model having highest R2 value of 0.991 and 1, respectively, and also predicting reasonable kinetic coefficient values.

## Background

Phenols are chemical compounds which have been categorized in the high priority of pollutants by environmental protection agency (USEPA) [1]. Phenols are toxic compound at very low concentrations [2, 3]. So, phenol should be effectively removed from phenolic wastewater before discharge into water bodies [4]. The presence of phenols in wastewater such as oil refineries, petrochemical plants, olive oil, pesticide production and oil field operations contain high soluble solids (TDS) [5, 6] and in a olive oil plant, wastewater is acidic, high salty [7]. The concentrations of phenol in refinery and petrochemical industries, are in the ranges of 6–500 and 2.8-1220 mg/ml, respectively [8]. Various methods such as physical and chemical ones exist for the removal of phenol from saline wastewater, but they often have some problem including high costs and also production of hazardous products [2, 5, 6, 9]. The environmental treatment of saline wastewater by means of physic-chemical approaches like reverse osmosis, ion exchange, electrocoagulation etc. is not cost-effective [10, 11].

Kinetic models which related to culture growth and substrate utilization in presence of some inhibitory materials are showed in Table 1: [12]. In this situation, Haldane model is applied for representing the growth in both lower and higher concentrations of inhibitory materials.

**Table 1:** Kinetic models for growth on the presence of inhibitory substrates (Rozich, Gaudy et al. [11])

μ = μ_max_. S ÷ [S+ K_s_ + (S_2_/K_i_)]	(1)
μ = μ_max_. S [1 + (S/K)] ÷ [S+ K_s_ + (S_2_/K_i_)]	(2)
μ = μ_max_. S ÷ [S+ K_s_ + (S_2_/K_i_)] [1 + (S/K_i_)]	(3)
μ = μ_max_. S exp. (−S/K_i_) ÷ (S + K_s_)	(4)
μ = μ_max_ [exp (−S/K_i_)] – [exp (−S/K_s_)]	(5)

Results of Gaudy study are shown with the above given equation [13]. Hamoda proposed a mathematical model based on Monod model making it possible to determine the kinetic coefficients of aerobic submerged attached growth reactor. Kinetic coefficients of a submerged attached growth systems designing the removal of organic carbon are determined by this model (equation 1) [14].

1 $$ \frac{\left({S}_i-{S}_e\right)}{X_e}=\frac{k_d}{Y}\;\frac{\mathrm{A}\overline{\mathrm{X}}}{QX_e}+\frac{1}{Y} $$

Primitive equation (equation 2) of Modified Stover- Kincannon model designed by Stover for RBC reactor in 1982, has been reported according to the following reaction [15]:

2 $$ \frac{dS}{dt}=\frac{Q\left({S}_0-{S}_e\right)}{V}=\frac{U_{\mathrm{max}}\left(\frac{QS_0}{A}\right)}{K_B+\left(\frac{QS_0}{A}\right)} $$

If we replace the area disc with the effective volume of the reactor, in that case, we will have the equation below (equation 3) [15]:

3 $$ \frac{dS}{dt}=\frac{Q\left({S}_0-{S}_e\right)}{V}=\frac{U_{\mathrm{max}}\left(\frac{QS_0}{A}\right)}{K_B+\left(\frac{QS_0}{A}\right)} $$

This equation can also be written as a linear response as follows (equation 4):

4 $$ \frac{V}{Q\left({S}_0-{S}_e\right)}=\frac{K_B}{U_{\mathrm{max}}}\times \frac{V}{QS_0}+\frac{1}{U_{\mathrm{max}}} $$

Hussain et al. performed some experiments to study the kinetic study of aerobic treatment of phenolic wastewater. Kinetics of phenol degradation has also been studied using Haldane model [16]. In addition, Sahariah and Chakraborty were studied the kinetic analysis of phenol, thiocyanate and ammonia-nitrogen removal in an anaerobic-anoxic-aerobic moving bed bioreactor system [17].

Christian et al. studied phenol removal (754 mg/l) by Sulfolobus Solfataricus and calculated the kinetic parameters [18]. In the study of modeling of phenol removal by using attached growth olive pulp bacteria, the kinetic coefficients of μ_max_, K_s_ and K_i_ were determined as 1.296 d^−1^, 19.23 mg/l,1571 mg/l, respectively [19]. In another research performed by Rozich et al. which phenol was treated by activated sludge, the range of μ_max_ was equivalent to 1.92- 8.64 d^−1^ (average:4.56 d^−1^) and amount of K_s_ was 75 mg/l [20]. Moreover, Yalcin et al. carried out a study on the removal of phenol from wastewater. They reported that the kinetic coefficients of μ_max_ and K_s_ were found 4.432 d^−1^ and 87.4 mg/l, respectively. Marques et al. investigated the attached growth biomass and substrate utilization rate in the moving-bed biofilm reactor**.** They found that no significant difference was observed between the obtained kinetic parameters with those reported in the literature. The organic load and the substrate utilization rates were around 0.8 kg biomass/kg inert carrier which is considered so high [21]. Juang et al. reported kinetics of degradation of phenol in saline solution through solvent extraction with degradation in a two-phase partitioning bioreactor. They found that phenol would potentially be degraded in saline solution and in the aqueous cell medium, although the initial phenol concentrations showed an increasing trend up to 1850 g/m^3^ [22]. Naghizadeh et al. studied the application of a Hollow- FiberMembrane in removal of COD, TNa and TP from wastewater and reported that the applied method can potentially be considered as an effective remediation method for different applications in wastewater effluent reuse [23]. Mahvi et al. were envestigated the Photo-oxidation of phenol in water media: Toxicity of intermediates [24]. Maleki et al. studied the degradation and toxicity reduction of phenol by ultrasound waves. In this study pH, kinetic constants and initial phenol concentration on the sonochemical degradation of phenol and toxicity assay were investigated [25]. Ahmadi et al. were used Kocuria turfanesis strain M7, Halomonas Alkaliphila strain R4, Pseudomonas balearica strain Z8 for treatment a high saline petrochemical wastewater, results of kinetic evaluation demonstrated that the yield (Y), endogenous decay coefficient (kd), maximum reaction rate (Kmax), maximum specific growth rate (mmax) and saturation constant (Ks) were 0.54 mg VSS mg COD^−1^, 0.014 day^−1^, 1.23 day^−1^, 0.66 day^−1^, and 1315 mg L^−1^, respectively [26]. This study was performed to help bridging this gap in knowledge, by evaluating phenol degradation by MBBR in saline solution and by determining corresponding kinetic rate parameters.

## Methods

### Reactor setup

**Fig. 1:**
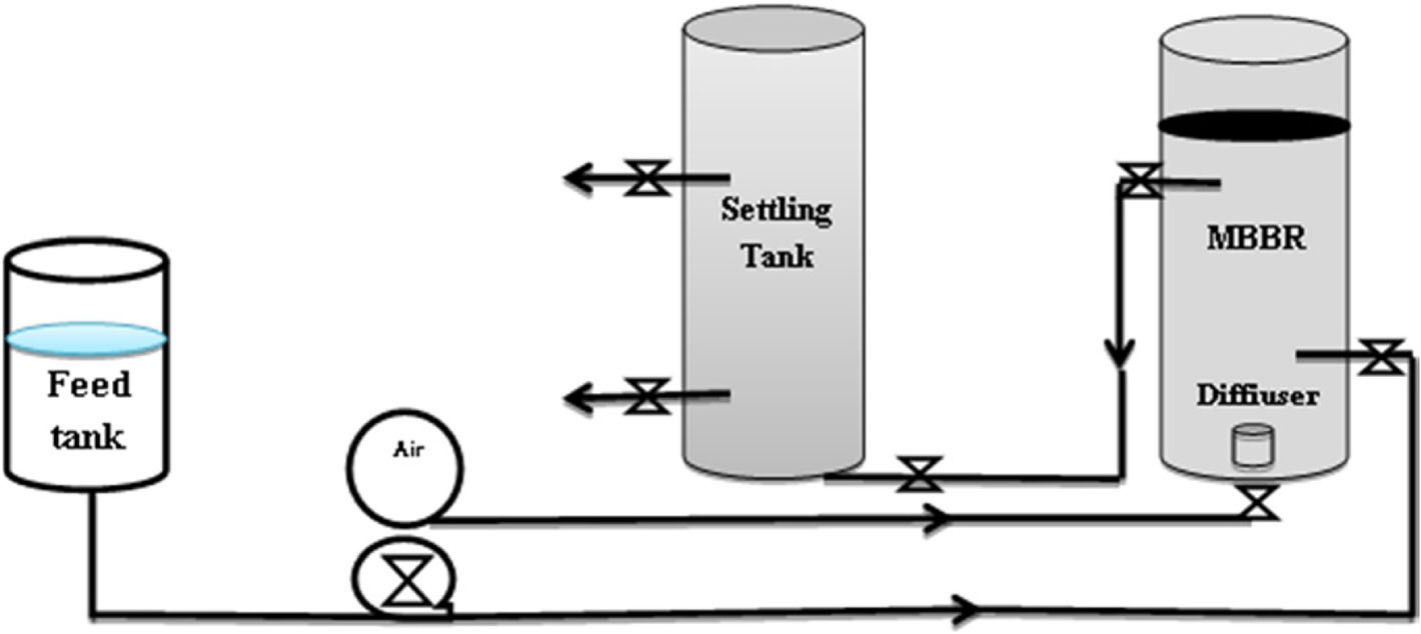
The schematic of the MBBR experimental set up

Tables 2 and 3 show MBBR and settling tank characteristics used in this study. Synthetic wastewater was fed by a peristaltic pump and the reactor was aerated using a stone air diffuser situated at the bottom of the reactor feed by the compressor. Figure 1 shows a schematic of MBBR used in this study. MBBR reactor was filled with polyethylene media characterized in Table 4; also, to avoid leaving the media from the reactor, a plate screen was installed on top of the reactor.

**Table 2:** Description of the MBBR reactors used in the pilot- scale

Characteristics (units)	Values
External diameter (mm)	160
Internal diameter (mm)	135
Total height (mm)	650
Internal height (mm)	635
Effective height (mm)	440
Total volume (L)	9.5
The volume occupied by the media (L)	3.2
Effective volume (L)	6.3

**Table 3:** Description of the settling tank used in the pilot- scale

Characteristics (units)	Values
External diameter (mm)	165
Internal diameter (mm)	135
Total height (mm)	350
Internal height (mm)	325
Effective height (mm)	210
Total volume (L)	9
Effective volume (L)	3

**Table 4:** Description of the media used in the pilot- scale

Characteristics	Values
Specific area (m^2^. Each)	18
Number in each m^2^	36,100
Specific surface area (m^2^.m^−3^)	650
Density (Kg.m^−3^)	140
Porosity (%)	87

### Reactor operation

The MBBR used in this work was operated with HRT of 6 h for 233 days. Reactor operation was started with 3 L of the municipal activated sludge and 3 L of synthetic wastewater containing acetate and essential nutrients. Municipal sludge characterization was evaluated in terms of mixed liquor suspended Solids (MLSS), sludge volume index (SVI) and microscopic assay; furthermore, microscopic assessment was done during experiments. The samples were also collected from influent and sampling port of each reactor. The applied synthetic wastewater consisted of acetate phenol as carbon source and nutrient containing dipotassium mono hydrogen phosphate, potassium dihydrogen phosphate, sodium bicarbonate, ammonium chloride, magnesium sulfate, calcium chloride, boric acid, ferric chloride, copper sulfate, Iodide potassium, chloride, manganese, zinc, cobalt chloride, and magnesium thiosulfate (Merck company, Germany) resulting in C/N/P as 100/5/1. In order to prevent decreasing pH, the alkalinity was enhanced through addition of sodium bicarbonate, pH value range of aerobic biological process was maintained and dissolved oxygen in the reactor was maintained at a range of 1–2 mg. In adaptation stage, phenol concentration showed an increasing during 120 days and simultaneously acetate decreased stepwise until phenol increased to 100% of the target amount and acetate reached to 0 mg/l. Effect of phenol concentration (0–500 mg/l) on the MBBR performance was also carried out in three phases 0–100, 100–300 and 300–500 mg/l, where phenol concentrations increased gradually during 105 days of reactor operation with TDS = 0. A total of 105 days of reactor operation comprised 15, 23 and 21 days for the three steps and 46 days between three steps respectively were attributed to evaluation of the effect of phenol concentration and effluent stability. Moreover, the effect of TDS concentration (0-3%) on the MBBR performance was evaluated during 114 days with a gradual increase in TDS in three phases 0–1, 1–2 and 2–3% with a constant concentration of 500 mg/l phenol. A total of 114 days of reactor operation comprised 18, 18 and 13 days of long operation for the three steps and 65 days between three steps respectively were attributed to evaluate the effect of TDS concentration and effluent stability. Seven days of constant phenol concentration in the effluent were defined as stability of the reactor.

### Microorganisms and culture acclimatization condition

The Moving-bed Biofilm Reactor in this study for the growth studies was performed with HRT of 6 h over a period of 233 days. Reactor was operated with activated sludge obtained from municipal wastewater treatment. The applied synthetic medium in the reactor for growth studies is reported in Table 5.

**Table 5:** Stock solutions used in growth medium solutions used

Compound	mg/l
K_2_HPo_4_	58
KH_2_Po_4_	25
CaC1_2_	50
MgSo_4_	75
Na_2_Co_3_	200
NH_4_Cl	191
H_3_Bo_3_	1500
Fecl_3_ .6H_2_o	150
CuSO_4_ .5H_2_O	30
MnCL_2_ .4H_2_O	30
ZnSO_4_ .7H_2_O	120
COCl_2_ .6H_2_O	60
Na_2_MnSO_4_ .2H_2_O	150

In adaptation stage, phenol concentration increased during 120 days and simultaneously acetate decreased stepwise so that phenol evaluated to 100% of the target amount and acetate reached 0 mg/l. Effect of phenol concentration (0–500 mg/l) over the MBBR performance conducted in three separate phases including 0–100, 100–300 and 300–500 mg/l. Phenol concentration witnessed a gradual increasing trend during 105 days of reactor operation with TDS = 0. A total 105 days of reactor operation consisted of 15, 23 and 21 days operation for the three steps and 46 days between three steps, respectively. They were attributed to the evaluation of effect of phenol concentration and effluent stability. Moreover, the effect of TDS concentration (0-3%) on the MBBR performance was evaluated during the 114 days with which showed a gradual increase in TDS in three phases 0–1, 1–2 and 2–3% with a constant phenol concentration of 500 mg/l. A total of 114 days of reactor operation consisted of 18, 18 and 13 days of long operation for the three steps and 65 days between three steps respectively were ascribed to the evaluation of effect of TDS concentration and effluent stability. Seven days of constant phenol concentration in the effluent were defined as stability of the reactor.

### Analytical procedure

During the period of continuous operation, some samples were obtained from the influent and effluent of the MBBR every day. The concentration of phenol was determined using spectrophotometry and by the colorimetric 4-aminoantipyren procedure as detailed in the Standard Methods (0.002 to 0.200 mg/L) [27] using a Spectrophotometer DR 5000 UV/VIS (HACH company, USA). TDS was also measured by means of a TDS meter (HACH company, USA) as well.

### Modeling the kinetics of phenol biodegradation

The principal aim of the present research was to define the growth and biodegradation kinetics of microorganisms at phenol concentration of 500 mg/l and salt concentration of 3% with HRT of 6, 12, 18, 24 and 36 h. For determination of the amount of biomass concentration at each stage, some number of media were removed randomly from the MBBR reactor which was followed by weighting. By measuring the weight difference of media with initial weight of media at each stage, biomass concentration was calculated. For conduction a comprehensive research, various kinetic models for degradation of phenol were applied. The kinetic parameters were also calculated according to Monod, Modified Stover- Kincannon, Hamoda and Haldane models. In Monod model, K and K_s_ were calculated by plotting of S-S_0_/X vs. 1/S, Y and K_d_ were calculated with plotting 1/S vs. S_0_-S/X. The Haldane equation is used (μ = μ_max_. S ÷ [S + K_s_ + (S_2_/K_i_)]) for determination of the inhibitory coefficient (K_i_). In Hamoda model, K and K_s_ were calculated by plotting 1/S vs. [A $\overline{\mathrm{X}}$ /Q (S_0_-S)]. The slope of regression line was equal to K/K_s_ and the intercept of regression line was equal to 1/K_s_ by plotting of [(S_0_-S)/X] vs. (A $\overline{\mathrm{X}}$ /QX), the slope of the regression line was equal to K_d_/Y and intercept was equal to 1/Y. The Modified Stover-kincannon were calculated K_B_ and U_max_ by plotting V/Q (S_0_ - S_e_) vs. V/QS_0_.

## Results

**Fig. 2:**
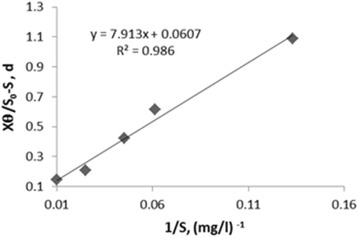
Regression between X/S_0_-S and 1/S to determine K_s_ and K using Monod model

The kinetic coefficients were determined at phenol concentration of 500 mg/l and salinity of 3%. A plot of 1/S vs. X/S_0_-S (Fig. 2) was also depicted for evaluation of K and K_s_ for the phenol biodegrading microorganisms, based on Monod equation. Therefore, the amounts of K and K_s_ were calculated as 16.47 d^−1^, 130.32 mg/l, respectively.

**Fig. 3:**
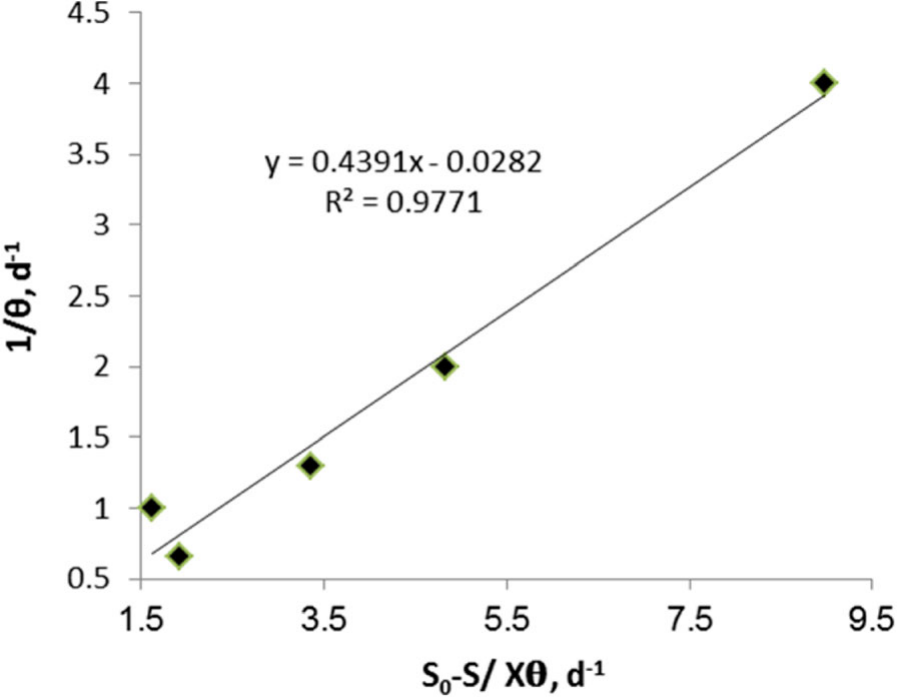
Regression between S_0_-S/X θ and 1/θ to determine Y and K_d_ using Monod model

**Fig. 4:**
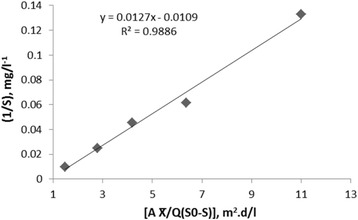
Regression between [A $\overline{\mathrm{X}}$ /Q(S0-S)] and 1/θ to determine K and K_s_ using Hamoda model

**Fig. 5:**
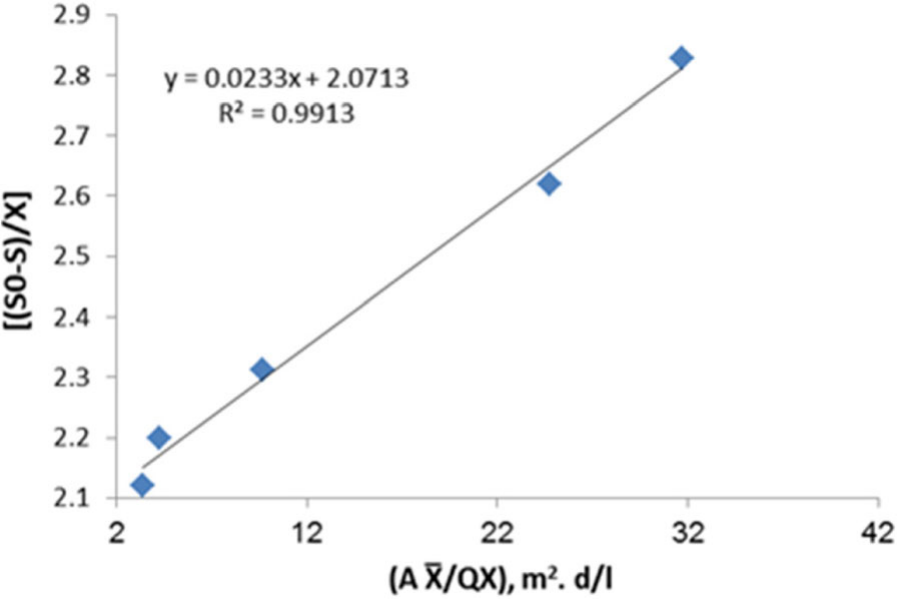
Regression between (A $\overline{\mathrm{X}}$ /QX) and [(S_0_-S)/X] to determine Y and K_d_ using Hamoda model

**Fig. 6:**
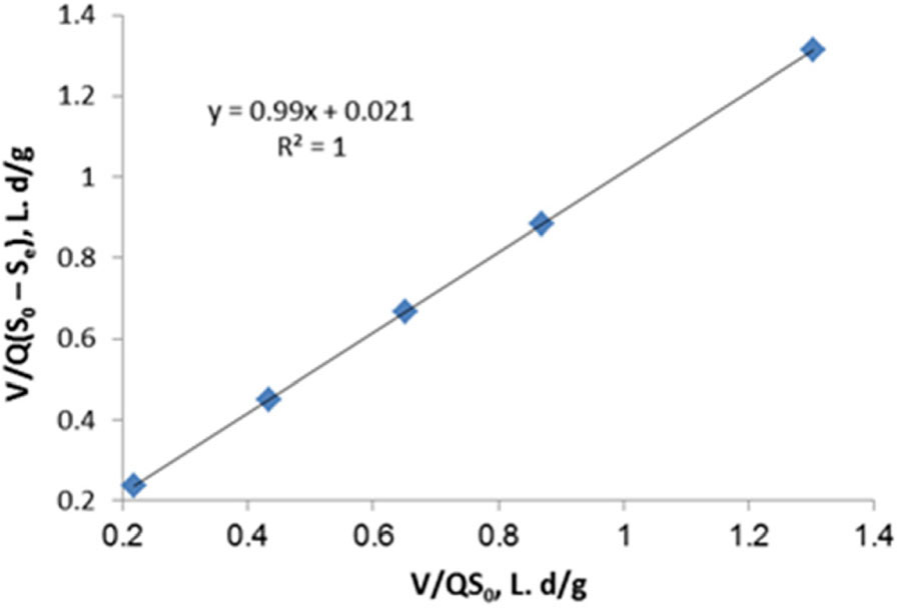
Determination of Kinetic coefficients of the modified Stover - Kincannon model

Assuming a specific decay rate, K_d_ of 0.0282 d^−1^ and Y of 0.4391 mg/mg were estimated by statistically fitting the observed data to an attached-growth Monod-type rate expression using linear regression techniques according to Fig. 3. The amount of K_i_ in Haldane equation, (μ = μ_max_. S ÷ [S + K_s_ + (S_2_/K_i_)]) was calculated as 1641 mg/l. The kinetic coefficients of K, K_s_, Y and K_d_ in Hamoda model, is shown in Figs. 4 and 5. The kinetic parameters of K, K_s_, Y and K_d_ were calculated 1.1 d^−1^, 91.74 mg/l, 0.482 mg/mg and 0.011 d^−1^, respectively. The kinetic parameters of U_max_ and K_B_ in modified Stover - Kincannon model, according to Fig. 6, were calculated 47.61 g/l.d and 13.47 g/l.d, respectively. This study was investigated the growth and biodegradation kinetics at 500 mg/l of phenol and salt concentration of 3% with HRT of 6, 12, 18, 24 and 36 h.

## Discussion

Kinetic studies are very important for industrial laboratory, in order to justify kinetic studies to generalize why using their results in the industrial scale [24]. In the study reported by Nadafi et al., the effect of lyophilized on the kinetic coefficients of the activated sludge was evaluated. The amounts of K, K_S_, K_d_, Y and μ_max_ were obtained as 3.2 d^−1^, 71.7 mg/l, 0.077 d^−1^, 0.48 mg/mg, 1.5 d^−1^, respectively [25]. Kinetic parameters such as K, K_s_, Y and K_d_ according to Hamoda model were calculated as 1.1 d^−1^, 9.74 mg/l, 0.479 mg/mg, 0.01 d^−1^. The kinetic coefficients of Modified Stover – Kincannon model, U_max_ and K_B_ were calculated, 47.61 g/l.d and 47.13 g/l.d, respectively. The amount of kinetic coefficients of K, K_s,_ K_d_, Y and μ_max_ in Monod model were calculated 47.16 d^−1^, 130.32 mg/l, 0.0282 d^−1^, 0.439 mg/mg and 7.23 d^−1^, respectively. The amount of the K_i_ according to Haldane equation, (μ = μ_max_. S ÷ [S + K_s_ + (S_2_/K_i_)]) was calculated 1641 mg/l. The growth kinetics parameter values obtained from different models are shown in Table 6.

**Table 6:** Summary of growth kinetics parameter values obtained from different models during biodegradation of phenol

Model	μ_max_(d^−1^)	K_S_(mg/l)	K (d^−1^)	K_d_(d^−1^)	K_i_(mg/l)	Y (mg/mg)	U_max_g/l.d	K_B_g/l.d
Monod	7.23	130.32	47.16	0.0282	–	0.439	–	–
Haldane	–	–	–	–	1641	–	–	–
Hamoda		9.74	1.1	0.479	–	0.01	–	–
Stover-Kincannon	–		––	–	–	–	47.61	47.13

Tallaei et al. studied the kinetic parameters of crude oil decomposition by means of pseudomonas aeruginosa bacteria, The amount of K, K_S_, K_d_ and Y were also determined as 9.39 mg/l, 169.3 mg/l, 0.1071 d^−1^, 0.882 mg/mg, respectively [28]. For finding the kinetic parameters of phenol in saline wastewater in high concentration of 50 mg/l, μ_max_, K and Y were calculated as 5.28- 9.36 d^−1^, (33.4- 27.7) and 0.19- 0.28 mg/mg, respectively. By increasing phenol concentration up to 300 mg/l, the amount of μ_max_, Y, K were decreased to 5.28- 2.16, 0.2-0.29, 10.2- 18.2, respectively [29]. Results of this study were similar to present study. Rozich et al., reported that phenol was treated by activated sludge, the range of μ_max_ and amount of K_s_ were 1.92-8.64 d^−1^ (by average: 4.56 d^−1^) and 75 mg/l in their investigation, respectively [19]. In the study by Yalcin et al., about phenol removal from wastewater, the amounts of μ_max_ and K_s_ were calculated as 4.432 d^−1^ and_,_ 87.4 mg/l, respectively. The findings of that research were different from present one [30]. In another study performed by Christian et al., the removal of phenol (754 mg/l) was found by Sulfolobus solfataricus, the quantities of μ, K_s_, K_i_ and μ_max_ were also calculated as 2.256 d^−1^, 77.7 mg/l, 319.4 mg/l and 1.128 d^−1^, respectively [17]. Moreover, in the study of modeling phenol by using attached growth olive pulp bacteria, μ_max_, K_s_, K_i_ were calculated as 1.296 d^−1^, 19.23 mg/l, 1571 mg/l, respectively. The inhibitor constant, in this study, was found 1641 mg/l [18]. The best model was selected for the evaluation process by evaluation of the correlation coefficients (*R* ^*2*^). According to Figs. 1 and 2, the correlation coefficients of Monod model were obtained 0.986 and 0.9771, respectively. According to Figs. 3 and 4, the correlation coefficients of Hamoda model were obtained 0.9886 and 0.9913, respectively. According to Fig. 5, the correlation coefficients of Modified Stover-Kincannon model were also equal to 1. Results revealed that the best models for fitting the experimental data of this study are Hamoda and Modified Stover- Kincannon models, having highest *R* ^*2*^ value and predicting reasonable kinetic coefficient values. The literature reported that performance of both Hamoda and Modified Stover-Kincannon models were more suitable for designing the aerobic submerged attached growth biological reactors [13, 31, 32].

## Conclusion

In this research, we studied the growth and biodegradation kinetics of microorganisms at 500 mg/l phenol concentration and salinity of 3%. According to Monod equation, K, K_s_, K_d_, Y and μ_max_ were calculated 16.147 d^−1^, 130.32 mg/l, 0.0282 d^−1^, 0.4391 mg/mg and 7.23 d^−1^, respectively. Kinetic parameters like K, K_s_, Y and K_d_, according to Hamoda model, were calculated as 1.1 d^−1^, 91.47 mg/l, 0.482 mg/mg, 0.011 d^−1^, respectively. Kinetic parameters of the modified Stover- kincanon model (U_max_ and K_B_) were calculated as 47.61 g/l.d and 13.47 g/l.d, respectively. The value of K_i_ in saline phenolic wastewater in attached growth system was higher than suspended growth systems which represented a higher phenol inhibition in suspended growth systems. Hamoda model and the Modified Stover-Kincannon model having highest *R* ^*2*^ value of 0.991 and 1, respectively, and also predicting reasonable kinetic coefficient values.

## Abbreviations

$$ \overline{X} $$: Attached biomass
A: Surface area of support media (m^2^)
HRT: Hydraulic retention time
K: Overall reaction rate (d^−1^)
K_B_: Saturation coefficient (g/L. d)
K_d_: Biomass decay rate (d^−1^)
K_i_: Inhibition coefficient (mg/l)
K_S_: Half saturation coefficient (mg/l)
MBBR: Moving bed biofilm reactor
Q: Flow rate (m^3^/d)
S: Phenol concentration (mg/l)
S_0_: Initial phenol concentration (mg/l)
t: Time (h)
TDS: Total dissolved solids
U_max_: Maximum substrate removal (g/L. d)
V: Volume of reactor (m^3^)
X: Microorganism concentration (mg/l)
X_0_: Initial microorganism concentration (mg/l)
Y: Yield coefficient (mg biomass/mg substrate)
μ: Specific growth rate (h^−1^)
μ_max_: Maximum specific growth rate (h^−1^)

## References

[1] USEPA (1985). Technical support document for water quality based toxics control, EPA/440/485032.

[2] Juang RS, Tsai SY (2006). Enhanced biodegradation of mixed phenol and sodium salicylate by pseudomonas putida in membrane contactors. Water Res.

[3] Hq L, Han H, Du M, Wang W (2011). Removal of phenols, thiocyanate and ammonium from coal gasification wastewater using moving bed biofilm reactor. Bioresource Tech.

[4] Dotto GL, Costa JAV, Pinto LAA (2013). Kinetic studies on the biosorption of phenol by nanoparticles from Spirulina sp. LEB 18. J Environ Chem Eng.

[5] Moussavi G, Khavanin A, Alizadeh R (2009). The investigation of catalytic ozonation and integrated catalytic ozonation/biological processes for the removal of phenol from saline wastewaters. Hazard Mat.

[6] Moussavi G, Barikbin B, Mahmoudi M (2010). The removal of high concentrations of phenol from saline wastewater using aerobic granular SBR. J Chem Eng.

[7] Aggelis G, Iconomou D, Christou M, Bokas D (2003). Phenolic removal in a model olive oil mill wastewater using Pleurotus ostreatus in bioreactor cultures and biological evaluation of the process. Water Res.

[8] Dosta J, Nieto JM, Vila J, Grifoll M, Mata-Álvarez J (2011). Phenol removal from hypersaline wastewaters in a membrane biological reactor (MBR): operation and microbiological characterisation. Bioresource Tech.

[9] Fang HHP, Liang DW, Zhang T, Liu Y (2006). Anaerobic treatment of phenol in wastewater under therm ophilic condition. Water Res.

[10] Abou-Elela SI, Kamel MM, Fawzy ME (2010). Biological treatment of saline wastewater using a salt-tolerant microorganism. Desalination.

[11] Rozich AF, Gaudy AF, D'adamo PC (1984). Selection of rate model for Actived Sludges treating phenol. Water Res.

[12] Ahmadi M, Amiri H, Martínez SS (2012). Treatment of phenol-formaldehyde resin manufacturing wastewater by the electrocoagulation process. Desalin Water Treat.

[13] Gaudy AF, Rozich AF, Gaudy ET (1986). Actived sludge process models for treatment of toxic and nontoxic wastes. Water Sci and Tech.

[14] Hamoda MF, Zeidan MO, Al-Haddad AA (1996). Biological nitrification kinetics in a fixed-film reactor. Bioresource Tech.

[15] Jin RC, Zheng P (2009). Kinetics of nitrogen removal in high rate anammox upflow filter. Hazard Mat.

[16] Hussain A, Dubey SK, Kumar V (2015). Kinetic study for aerobic treatment of phenolic wastewater. J water Resours.

[17] Sahariah BP, CaaKraborty SN (2011). Kinetic analysis of phenol, thiocyanate and ammonia-nitrogen removals in an anaerobic–anoxic–aerobic moving bed bioreactor system. J Hazard Mater.

[18] Christen P, Vega A, Casalot L, Simon G, Auria R (2012). Kinetics of aerobic phenol biodegradation by the acidophilic and hyperthermophilic archaeon Sulfolobus solfataricus 98/2. J Biochem Eng.

[19] Tziotzios G, Lyberatos G, Pavlou S, Vayenas DV (2008). Modelling of biological phenol removal in draw-fill reactors using suspended and attached growth olive pulp bacteria. Int Biodeter & Biodeg.

[20] Marques JJ, Souza RR, Souza CS, ICC R (2008). Attached biomass growth and substrate utilization rate in a moving bed biofilm reactor. Brazillian J Chem Eng.

[21] Juang RS, Kao HC, Tseng KJ (2010). Kinetics of phenol removal from saline solutions by solvent extraction coupled with degradation in a two-phase partitioning bioreactor. Separ and Purifi Tech.

[22] Kumar A, Kumar S, Kumar S (2005). Biodegradation kinetics of phenol and catechol using pseudomonas putida MTCC 1194. Biochem Eng J.

[23] Naghizadeh A, Mahvi A, Vaezi F, Naddafi K (2008). Evaluation of hollow fiber membrane bioreactor efficiency for municipal wastewater treatment. J Environ Health Sci Eng.

[24] Mahvi AH, Maleki A, Alimohamadi M, Ghasri A (2007). Photo-oxidation of phenol in aqueous solution: toxicity of intermediates. Korean J Chem Eng.

[25] Maleki A, Mahvi AH, Mesdaghinia A, Naddafi K (2007). Degredation and Toxity reduction of phenol by ultrasound Wawes. Chem Soci Ethiopia.

[26] Ahmadi M, Jorfi S, Kujlu R, Ghafari S, Soltani R, Jaafarzadeh N. A novel salt-tolerant bacterial consortium for biodegradation of saline and recalcitrant petrochemical wastewater. J Environ Manag. 2017;191:198–208. 10.1016/j.jenvman.2017.01.010 28104552

[27] APHA, AWWA, WPCF. Standard methods for the examination of water & wastewater washington, DC, USA; 2005.

[28] Tallaei A, Jaafarzadeh N, Tallaei MR, Beheshti M. Determibnation of kinetic parameters of crude oil by using Pseudomonas aeruginosa bacteria. J Environ Health. 2010;3:111–22.

[29] Peyton BM, Wilson T, Yonge DR (2002). Kinetics of phenol biodegradation in high salt solutions. Water Rese.

[30] Nuhoglu A, Yalcin B (2005). Modelling of phenol removal in a batch reactor. Process Biochem.

[31] Deshpande AM, Satyanarayan S, Ramakant (2012). Kinetic analysis of an anaerobic fixed-film fixed bed-reactor treating wastewater arising from production of a chemically synthesized pharmaceutical. Environ Science and Tech.

[32] Borghei SM, Sharbatmaleki M, Pourrezaie P, Borghei G (2008). Kinetics of organic removal in fixed-bed aerobic biological reactor. Bioresource Tech.

